# Performance of cone-beam computed tomography (CBCT) in comparison to conventional computed tomography (CT) and magnetic resonance imaging (MRI) for the detection of bone invasion in oral squamous cell cancer (OSCC): a prospective study

**DOI:** 10.1186/s12903-024-04057-4

**Published:** 2024-03-16

**Authors:** Anton Straub, Christian Linz, Constantin Lapa, Stefan Hartmann, Alexander C. Kübler, Urs D. A. Müller-Richter, Julian Faber, Thorsten Bley, Joachim Brumberg, Olivia Kertels, Roman C. Brands

**Affiliations:** 1 Department of Oral and Maxillofacial Plastic Surgery, University Hospital Würzburg, Pleicherwall 2, 97070 Würzburg, Germany; 2 Department of Oral, Maxillofacial and Plastic Surgery, University of Köln, Faculty of Medicine and University Hospital Köln, Kepenerstr. 62, 50937 Köln, Germany; 3 Nuclear Medicine, Faculty of Medicine, University of Augsburg, Stenglinstr. 2, 86156 Augsburg, Germany; 4 Department of Diagnostic and Interventional Radiology, University Hospital Würzburg, Oberdürrbacherstr. 6, 97080 Würzburg, Germany; 5 Department of Nuclear Medicine, Medical Center, Faculty of Medicine, University of Freiburg, University of Freiburg, Hugstetterstr. 55, 79106 Freiburg, Germany; 6 Department of Diagnostic and Interventional Neuroradiology, Klinikum rechts der Isar, Technical University of München, Ismaninger Str. 22, 81675 München, Germany

**Keywords:** Bone invasion, Bone resection, Computed tomography, Cone-beam computed tomography, Head and neck cancer, Mandibulectomy, Magnetic resonance imaging, Oral cancer, Oral squamous cell carcinoma

## Abstract

**Background:**

Oral squamous carcinoma (OSCC) is often diagnosed at late stages and bone erosion or invasion of the jawbone is frequently present. Computed tomography (CT) and magnetic resonance imaging (MRI) are known to have high diagnostic sensitivities, specificities, and accuracies in detecting these bone affections in patients suffering from OSCC. To date, the existing data regarding the impact of cone-beam computed tomography (CBCT) have been weak. Therefore, this study aimed to investigate whether CBCT is a suitable tool to detect bone erosion or invasion in patients with OSCC.

**Methods:**

We investigated in a prospective trial the impact of CBCT in the diagnosis of bone erosion or invasion in patients with OSCC who underwent surgery. Every participant received a CBCT, CT, and MRI scan during staging. Imaging modalities were evaluated by two specialists in oral and maxillofacial surgery (CBCT) and two specialists in radiology (CT and MRI) in a blinded way, to determine whether a bone affection was present or not. Reporting used the following 3-point system: no bony destruction (“0”), cortical bone erosion (“1”), or medullary bone invasion (“2”). Histological examination or a follow-up served to calculate the sensitivities, specificities, and accuracies of the imaging modalities.

**Results:**

Our results revealed high diagnostic sensitivities (95.6%, 84.4%, and 88.9%), specificities (87.0%, 91.7%, and 91.7%), and accuracies (89.5%, 89.5%, and 90.8%) for CBCT, CT, and MRI. A pairwise comparison found no statistical difference between CBCT, CT, and MRI.

**Conclusion:**

Our data support the routine use of CBCT in the diagnosis of bone erosion and invasion in patients with OSCC as diagnostic accuracy is equal to CT and MRI, the procedure is cost-effective, and it can be performed during initial contact with the patient.

## Introduction

Oral squamous cell carcinoma (OSCC) accounts for approximately 5% of all malignant tumors and occurs more frequently in men than in women. The diagnosis is often made at advanced disease stages, and consequently, infiltration of the maxillary and mandibular bone is frequently present in OSCC [1–3]. Despite treatment options such as microvascular reconstructive surgery or newly approved immunotherapies, the five-year survival rate is rather poor and has increased from 55 to only 66% within the last three decades [4]. In addition to classic risk factors such as alcohol and tobacco consumption, infection with human papilloma (HP) viruses or chewing areca nut products play an important role [5, 6]. However, the different causes of tumorigenesis do not lead to different recommendations regarding the diagnosis and therapy of OSCC [2].

Most commonly, OSCC is treated surgically, with adjuvant radiation or combined radio-/chemotherapy depending on the tumor stage [7]. The presence of bone invasion leads to stage IVa status in the tumor, (lymph) nodes, metastases (TNM) classification, whereas only superficial erosion of the jawbone has no effect on the tumor stage. The distinction between erosion and invasion is not only reflected in the tumor stage but is also a prognostic factor and important for surgical planning and the decision whether adjuvant therapy is necessary [8, 9]. Bone erosion and especially invasion markedly increase the surgical requirement for resection and reconstruction. Therefore, bone erosion, for example, through OSCC of the alveolar gingiva in the lower jaw, could be treated with marginal mandibulectomy, while bone invasion usually requires segmental resection of the mandibula [8, 10]. After maxillary and mandibular bone removal, calvaria, iliac crest, and vascularized bone grafts are described in the literature for reconstruction. Iliac crest can be obtained as a non-vascularized bone graft or as a free microvascular flap. Furthermore, fibula or scapula flaps with or without a skin paddle are frequently used in reconstructive maxillofacial surgery [11–14]. For surgical planning, it is therefore crucial not only to detect the presence of a bone affection preoperatively but also to assess its extent accurately (erosion vs. invasion) [10, 15].

The detection of bone affections caused by OSCC is normally performed with computed tomography (CT) or magnetic resonance imaging (MRI). Both radiological modalities can detect bone erosion or invasion with similar sensitivity, specificity, and accuracy. In the literature, these values were between 41.7 and 89, 86.9–100, and 71.2–85 for CT and 58.3–95%, 73–100%, and 75.8–93%, respectively, for MRI [16–18]. Another option to illustrate bone structures and therefore detect bone erosion or invasion in patients with OSCC is cone-beam computed tomography (CBCT), which is commonly used in oral and maxillofacial surgery [19–21]. In particular, high resolution (0.09–0.4 mm), easy availability, and low radiation doses (29–477 µSv) are advantages of CBCT compared to CT and MRI [22, 23]. In the literature, CBCT has been reported to detect bone erosion and invasion with high sensitivity, specificity, and accuracy (Table 1) [16–18, 21, 24–27].

**Table 1:** Sensitivity, specificity and accuracy of CBCT in the detection of a bone invasion in the jaw bone [18, 21, 25, 27–30]

	Subjects	Sensitivity in %	Specificity in %	Accuracy (95% CI) in %
Wang et al., 2021	30	100	100	100
Czerwonka et al., 2017	45	91	86	--
Linz et al., 2015	197	87.9	83.2	84.8
Hakim et al., 2014	48	93	62	77
Dreiseidler et al., 2011	77	92	96.5	93.1
Hendrikx et al., 2010	23	90.9	100	95.7
Momin et al., 2009	50	89	60	--

The current literature has many limitations, such as small and heterogeneous study collectives, different scan protocols (e.g. slice thicknesses), or the fact that not all imaging modalities were performed in one patient; therefore, no intraindividual comparison of the CBCT, CT, and MRI scans was possible. This is the reason why the current German guidelines for the diagnosis and therapy of OSCC do not give a clear recommendation on which imaging modality should be used to detect bone erosion or invasion.

We hypothesize that CBCT is equally as reliable as CT and MRI in the detection of bone erosion and invasion in patients with OSCC. To our knowledge, this is the first prospective study to investigate the sensitivity, specificity, and accuracy of CBCT, CT, and MRI intraindividually in a large and consistent study collective with OSCC.

## Materials and methods

**Fig. 1:**
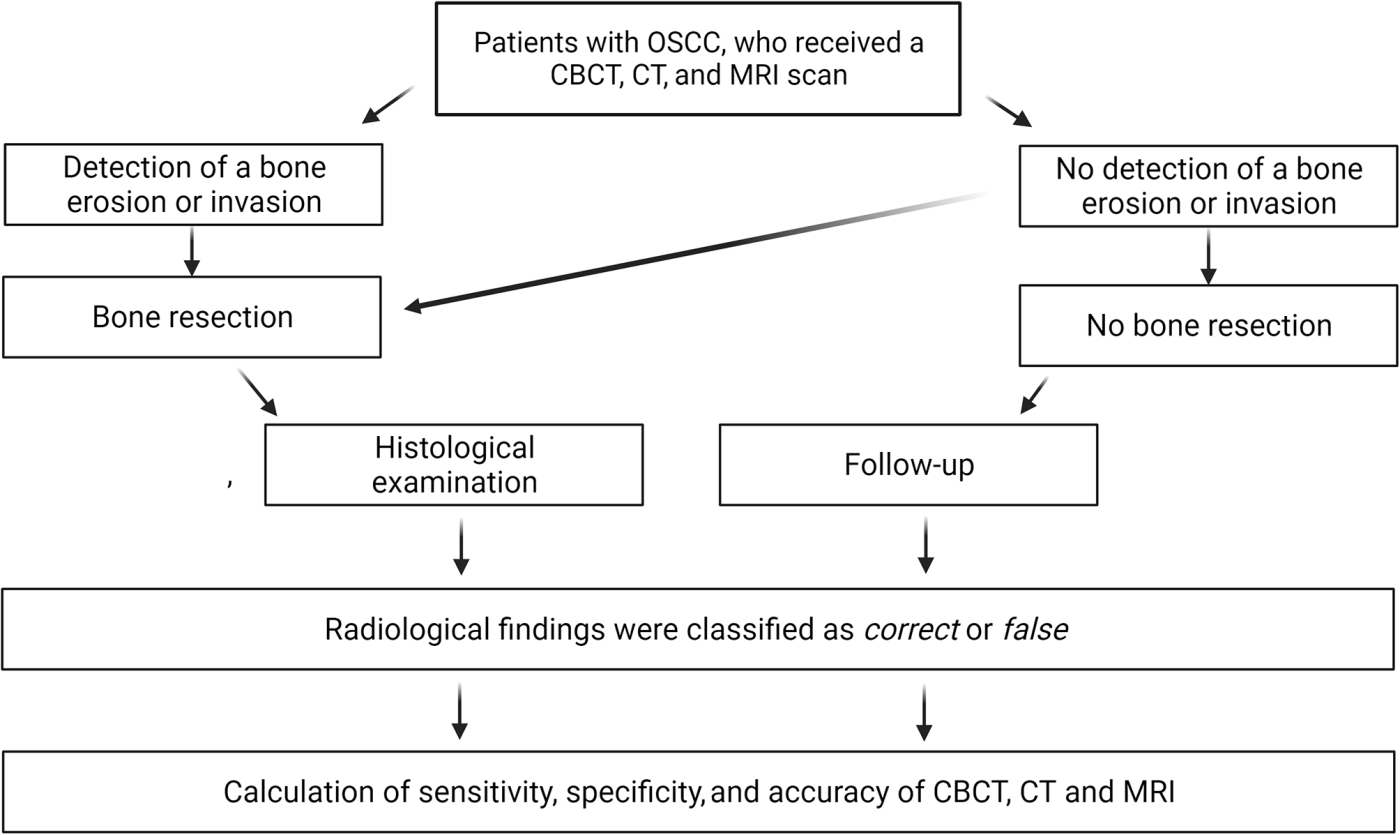
Flowchart of the study design. When bone erosion or invasion was detected on CBCT, CT, or MRI, a bone resection was performed. The imaging modalities were validated by histological examination of the bone tissue. When no bone erosion or invasion was detected in CBCT, CT, and MRI, normally no bone resection was performed. However, when the tumor had direct contact with the jawbone, a bone resection was performed in some cases, and a histological examination could serve to validate CBCT, CT, and MRI. In cases without any bone resection, a follow-up served to validate the radiological findings

We initiated a prospective trial from April 2013 to May 2016, in which we investigated the sensitivity, specificity, and accuracy of CBCT, CT, and MRI to detect bone erosion and invasion in patients with OSCC (Fig. 1).

The inclusion criteria were as follows:

- Age over 18.
- Histologically confirmed diagnosis of OSCC.
- No previous bone operations.
- Preoperative CBCT, CT, and MRI scan performed.
- Absence of foreign metal objects.
- Surgical tumor therapy administered no later than two weeks after completion of the staging examinations.
- Histological examination of the resected bone tissue or clinical and radiological follow-up for at least six months.

The exclusion criteria were as follows:

- Artifacts, which highly influence CBCT, CT, and MRI evaluations.
- Pre-existing osteoradionecrosis.
- Pre-existing drug-related osteonecrosis of the jaw.
- Malignancies other than OSCC.
- No histological examination or incomplete follow-up.

The institutional review board of the University of Würzburg approved all the protocols implemented in this study (IRB approval number: 286/12).

### Cone-beam computed tomography (CBCT)

CBCT was performed with a Sirona Dentsply Galileos device (Sirona Dental Systems Inc., Bensheim, Hesse, Germany). The CBCT scans were saved as DICOM files and had 512 × 512 × 512 voxels with a resolution of 0.29 mm. The field of view was 147 × 147 × 147 mm. The evaluation of the CBCT images was performed at a workstation with Picture Archiving and Communication System (PACS)-based software (Merlin, Version Rev: 5.4.168314).

**Fig. 2:**
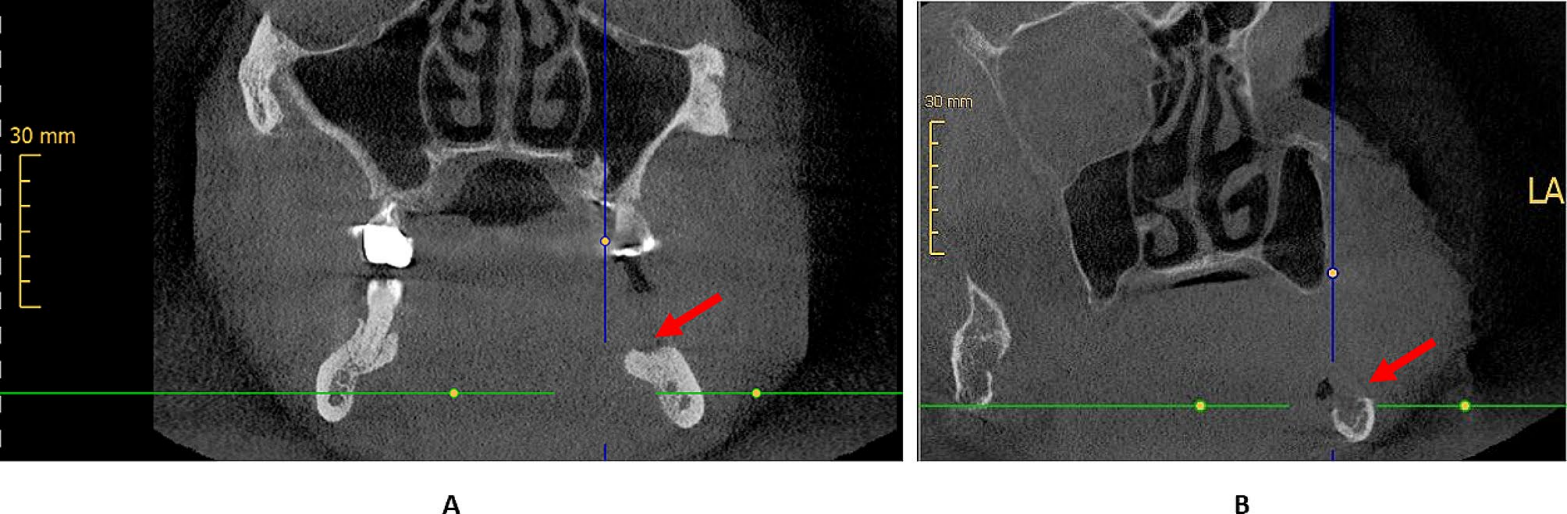
Panel A portrays a cortical erosion caused by an OSCC (red arrow), and Panel B portrays bone invasion (red arrow) of an OSCC in a different patient in a CBCT scan. A was thus scored in the 3-point system with a *1* (bone erosion), and B was scored with a *2* (bone invasion). The radiological findings in both cases were confirmed by histological examination

The evaluation was performed by two experienced specialists in oral and maxillofacial plastic surgery with an additional qualification in dental radiology, especially focusing on CBCT scans in consensus under standardized conditions at the same workstation and in a blinded manner (without knowledge of the reports of the other images and whether a bone invasion was present or not). Consensus means that both specialists agreed on the evaluation or discussed the findings until they reached a consent decision together. Information on the tumor location and any relevant previous surgery (for example, tumor surgery, decortication, or tooth extractions) was provided to the reporting physicians. Reporting used the following 3-point system: no bony destruction (“0”), bone erosion (“1”), or bone invasion (“2”) (Fig. 2). In addition, it was documented whether artifacts (for example, due to patient movements, dentures, foreign bodies, or similar instances) were present and whether these influenced the findings of bone integrity in the tumor area.

### Computed tomography (CT)

CT was performed with a PET/CT scanner (Siemens Biograph mCT 64, Siemens Healthineers, Knoxville, USA). Contrast-enhanced images from the thorax and abdomen were acquired (dose modulation with 180 mAs, 120 kV, 512 × 512 matrix, 5 mm slice thickness, increment of 30 mm/s, rotation time 0.5 s, and a pitch index of 1.4). Furthermore, a dedicated image of the head and neck region was performed using contrast-enhanced CT (180 mAs, 120 kV, 512 × 512 matrix, 0.75-5 mm slice thickness, increment of 30 mm/s, rotation time of 1.0 s, and a pitch index of 0.9).

### Magnetic resonance imaging (MRI)

MRI images were obtained from the head and neck region using a 1.5 T scanner (Siemens Magnetom Avanto fit, Siemens Healthcare, Erlangen, Germany) or a 3.0 T scanner (Magnetom Prisma or Skyra Siemens Healthcare, Erlangen, Germany) with a 20-channel head and neck coil as a signal receiver. MRI sequences included coronary T2-weighted inversion recovery magnitude (TIRM), axial T2-weighted sequences, coronary T1-weighted sequences with and without contrast enhancement and subtraction, axial T1-weighted sequences with fatty saturation and contrast enhancement, and axial diffusion weighting. A contrast agent (gadoterate-meglumine, dose 0.1 mmol/kg) was applied to patients using an injection system (Spectris MR Injector, Medrad) with a flow rate of 3 ml/second, followed by 20 ml of 0.9% saline. The MRI slice thicknesses were 3–4 mm.

### Image evaluation of CT and MRI scans

The evaluation was performed by two experienced specialists in radiology in consensus under standardized conditions at the same workstation and in a blinded manner (without knowledge of the reports of the other images and whether a bone invasion was present or not). Information on the tumor location and any relevant previous surgery (for example, tumor surgery, decortication, or tooth extractions) was provided to the reporting physicians. Reporting used the following 3-point system: no bone erosion or invasion (“0”), bone erosion (“1”), or bone invasion (“2”). In addition, it was documented whether artifacts (for example, due to patient movements, dentures, foreign bodies, or similar) were present and whether these influenced the findings of bone integrity in the tumor area (Fig. 2).

### Verification of CBCT, CT, and MRI

The CBCT, CT, and MRI reports were validated either with the result of the histological examination of the bone tissue or, if no bone tissue was harvested in the operation, by a clinical and radiological follow-up.

In general, bone was resected when the images detected bone erosion or invasion. In these cases, the histological examination validated the image reports. The bone tissue was fixed in formalin. Thereafter, a decalcification process with methanoic acid (formic acid) was performed for at least 72 h. Subsequently, the paraffin-embedded tissue was cut into 2 μm thick layers and stained with hematoxylin/eosin (HE).

Erosion was defined as an infiltration of the bone that is limited to the cortical bone. In contrast, invasion was defined as infiltration into the medullary cavity. Histological examination was performed under the supervision of a board-certified pathologist and served as the gold standard for verification of the imaging modalities.

When no bone tissue was obtained for a histological examination, a clinical and radiological follow-up served to verify the imaging findings. This follow-up was performed for at least six months and included a clinical examination and at least one postoperative CT or MRI scan. If no bone invasion was detected in the primary staging investigation by CBCT, CT, and MRI and a bone relapse occurred during the follow-up, the radiological findings were classified as false-negative when an operation followed and the histological examination confirmed the relapse. If no relapse was detected during the follow-up, the radiological findings were classified as correct-negative.

Furthermore, we performed different subgroup analyses to evaluate the effect of slice thicknesses in CT and to calculate the sensitivity, specificity, and accuracy only in patients with a histological examination of bone tissue (gold standard).

### Statistical analysis

Sensitivity, specificity, and accuracy were calculated for each of the above imaging modalities. Pairwise comparisons of the sensitivity and specificity of CBCT, CT, and MRI were performed using McNemar’s tests. All statistical tests were two-sided and calculated at a significance level of *p* < 0.05. No correction of *p* values was applied.

Statistical analysis was performed using R software (version 3.6.1, R Core Team, Vienna 2019) with Caret (version 6.0–84), DTComPair (version 1.0.3), pROC (version 1.15.3), and verification (version 1.42) packages.

## Results

Statistical analysis of all 153 patients revealed a mean age of approximately 64 years. The youngest patient was 23 years old, and the oldest was 94 years old. Eighty-seven of the 153 patients (57%) were male, and 66 were female. The median histological tumor size was 2.70 cm. T1 and T2 tumors were the most common, numbering 95 cases; T3 tumors were diagnosed in ten cases; and T4 tumors were diagnosed in 47 cases. Other tumor characteristics, such as lymph node status, grade, and venous, lymphatic vessel, and perineural sheath infiltration, are portrayed in Table 2.

**Table 2:** Categorical data

**Age (years)** range	63.6 ± 12.2 23–94
**Sex (n)**	
Male/female	87/66
**Localisation (n)**	
Right	65
Left	60
Anterior	21
Affecting right, left, and anterior region	7
**Surgical procedure (n)**	
No bone resection	76
Bone resection	77
Partial mandibular resection	36
Mandibulectomy	24
Maxillary partial resection	17
**Reconstruction (n)**	
Local or stalked flap	41
Microvascular radialis graft	85
Microvascular fibula graft	10
Other microvascular graft*	15
No information	2
**Adjuvant therapy (n)**	
No further therapy	49
RCT	64
RT	35
Not specified	5
**Histological results (n)**	
T1/T2/T3/T4/Tx	47/48/10/47/1
Mean tumor size	2.68 ± 1.42
N0/N1/N2a/N2b/N2c/N3/Nx	83/20/0/33/5/0/12
G1/G2/G3/Gx	18/99/33/3
Positive V/L/Pn	6/25/26
R0/R1/R2/Rx**	119/18/0/16
UICC stadium I/II/III/IVa/IVb	37/29/14/69/4
Detected bone invasion	45
**Mean duration of follow-up (months)**	27

RCT = radiochemotherapy, RT = radiotherapy alone, T1-4 = tumor size, N0-3 = nodal status, M = presence of distant metastases, G = grading, V = invasion into veins, L = invasion into lymphatic vessels, Pn = invasion into adjunct nerves, R0 = no residual tumor, R1 = microscopic residual tumor, R2 = macroscopic residual tumor, Rx = presence of a residual tumor cannot be assessed. *Other microvascular grafts were obtained from the scapula, vastus, anterolateral thigh, or soleus perforator flap. In two patients, no information was found about the reconstruction method that was used. **The data refer to the primary classification of the pathologist. Some patients who were classified as R1 received a second tumor resection and were finally classified as R0. Furthermore, some of the Rx patients were classified as R0 by the operator.

### Detection of a bone invasion

Across the entire cohort, a sensitivity of 95.6%, a specificity of 87.0%, and an accuracy of 89.5% (83.6–93.9%) with *p* < 0.001 (binominal test of the accuracy) were calculated for CBCT. For CT, a sensitivity of 84.4%, a specificity of 91.7%, and an accuracy of 89.5% (83.6–93.9%) were calculated with *p* < 0.001 (binominal test of the accuracy). For MRI, a sensitivity of 88.9%, a specificity of 91.7%, and an accuracy of 90.8% (85.1–94.9%) were calculated with *p* < 0.001 ((binominal test of the accuracy, Table 3). A pairwise comparison of the sensitivities and specificities of CBCT, CT, and MRI revealed no significant differences in these values (Table 4). The sensitivities of CBCT and CT did not differ significantly (*p* = 0.059) with one misclassification of CBCT when CT classification was correct and six misclassifications of CT when CBCT classification was correct (see Table 4, ^**+**^).

**Table 3:** General quality of the imaging modalities

	Sensitivity in %	Specificity in %	Accuracy (95% CI) in %	*p* value*
CBCT	95.6	87.0	89.5 (83.6–93.9)	< 0.001
CT	84.4	91.7	89.5 (83.6–93.3)	< 0.001
MRT	88.9	91.7	90.8 (85.1–94.9)	< 0.001

CBCT = cone-beam computed tomography, CT = computed tomography, MRI = magnetic resonance imaging. * Binominal test of the accuracy

**Table 4:**
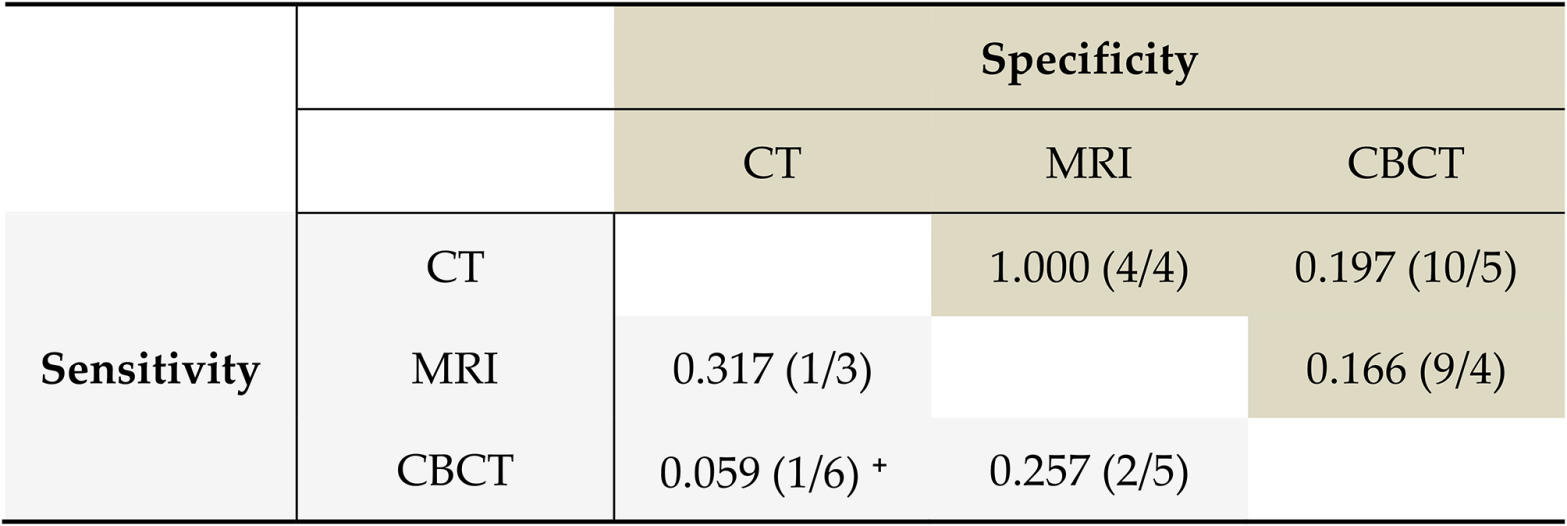
Pairwise comparisons of sensitivity and specificity

McNemar, *p*-values and in brackets number of misclassifications per procedure, CBCT = cone-beam computed tomography, CT = computed tomography, MRI = magnetic resonance imaging. In the grey area (specificity) the first value refers to the column, the second to the row. In the white area (sensitivity) the first value refers to the row, the second to the column

^**+**^ The sensitivity of CBCT and CT did not differ significantly (*p* = 0.059) with one misclassification of CBCT when CT classification was correct and six misclassifications of CT when CBCT classification was correct

### Comparison of the different slice thicknesses in CT

In subgroup A (CT slice thickness from 0.75 to 1.5 mm, *n* = 12), a sensitivity of 88.9%, a specificity of 66.7%, and an accuracy of 83.3% (51.6–97.9%) with *p* < 0.001 (binominal test of the accuracy) were calculated. In subgroup B (CT slice thickness 3 mm, *n* = 41), CT had a sensitivity of 83.3%, a specificity of 89.7%, and an accuracy of 87.8% (73.8–95.9%) with *p* < 0.009, and in subgroup C (CT slice thickness 4 and 5 mm, *n* = 100), a sensitivity of 83.3%, 93.4% and 91.0% (83.6–95.8%) with *p* < 0.001 was determined. No significant difference between CBCT and CT (and MRI) could be found in any subgroup.

### The subgroup with histological examination of bone tissue

Histological examination of resected bone tissue was conducted in 77 of the 153 patients. Histological examination is considered the gold standard to verify CBCT, CT, and MRI findings. For CBCT, a sensitivity of 95.6%, a specificity of 68.8%, and an accuracy of 84.4% were determined. For CT and MRI, the sensitivity was 84.4% and 88.9%, the specificity was 81.2% (for both modalities), and the accuracy was 83.1% and 85.7%. Differences between the imaging modalities were not statistically significant.

### Artifacts in CBCT, CT, and MRI

The evaluation of the frequency of artifacts interfering with image evaluation revealed no significant difference between CBCT and MRI. CT resulted in significantly more interfering artifacts than CBCT and MRI (Table 5).

**Table 5:** Comparison of the frequency of interfering artifacts between CBCT, CT, and MRI

		CT		MRI	
		No artifacts	Interfering artifacts	No artifacts	Interfering artifacts
CBCT	No artifacts	96	37	117	16
	Interfering artifacts	11	9	14	6
			< 0.001		0.855

McNemar, CBCT = cone-beam computed tomography, CT = computed tomography, MRI = magnetic resonance imaging. Portrayed are absolute numbers of the collective (*n* = 153).

## Discussion

CBCT is commonly used in oral and maxillofacial surgery, for example, in fracture diagnosis, implant planning, or in general, for therapy planning of jaw pathologies such as cysts [23]. Furthermore, some studies have shown evidence that CBCT detects bone invasion caused by oral malignancies with a high sensitivity, specificity, and accuracy. For example, Bombeccari et al. provided a literature overview evaluating seven studies that investigated the impact of CBCT in the detection of bone invasion in patients with OSCC. The diagnostic accuracy and negative predictive value of CBCT were high and comparable to those of CT and MRI. However, it was concluded that the available data are weak and that the impact of CBCT in the diagnosis of bone invasion remains unclear [17]. Studies investigating the impact of CBCT enrolled fewer than 50 patients, did not perform all radiological modalities in the same patient, or had a retrospective design [16–18, 21, 24–27]. One study enrolled 77 patients but only performed CBCT and CT scans. In that study, CBCT was similarly accurate to CT (sensitivity 92% versus 80% and specificity 96.5% versus 100%) [21]. Our study was designed to verify the value of CBCT in the detection of bone erosion or invasion and its role as a staging tool in patients with OSCC. Every patient who was included in our study preoperatively underwent a CBCT, CT, and MRI scan, allowing intraindividual assessment of these imaging modalities.

The sensitivites, specificities, and accuracies calculated in our study had similar values to those described in the literature and were high for all three imaging modalities without any significant differences among CBCT, CT, and MRI. Our results were highly significant (*p* < 0.001), and observer bias was eliminated by having every modality evaluated by two specialists in consensus. The sensitivity of CBCT was very high (95.6%), which is in line with the literature findings and plausible due to the high bone resolution of CBCT [31]. The specificity of CBCT was 87%, which was slightly lower than that of CT and MRI. The tumor itself cannot be visualized in a CBCT scan due to the lack of soft tissue imaging; therefore, it is conclusive that CBCT detects bone invasion with a high sensitivity but lower specificity. Furthermore, dental pathologies such as periodontitis can also lead to bone destruction and may result in higher false-positive results in CBCT, as reported in the literature and supported by the results of our study [30, 32]. The lack of soft tissue and tumor imaging could be responsible for this. A comparison of our findings with the literature is difficult because values are subject to large variations due to small study populations, the heterogeneity of study designs, and different scan protocols and imaging devices [21, 25, 27, 29].

CT and MRI image quality depends on the scan protocols and differs between studies. For example, image quality depends on the slice thicknesses, which were between 1.5 and 6 mm for CT and between 3 and 6 mm for MRI in most studies [33–35]. In our study, MRI slices were 3 mm in all patients. CT slices differ between 0.75 and 5 mm; we therefore performed a subgroup analysis to investigate the impact of different slice thicknesses in CT on the detection of bone erosion or invasion. Our results revealed no statistical significance between CBCT and CT for small (0.75–1.5 and 3 mm) and large (4–5 mm) slice thicknesses. Values in a small study cohort are subject to large variations, which limits their reliability. It is possible that larger study collectives would find a benefit in favor of thin CT slices in the detection of bone erosion or invasion.

Furthermore, we performed a subgroup analysis considering only patients with a histological examination of resected bone tissue. This is important because this specific histological examination is considered as the gold standard in the detection of bone erosion or invasion and for the distinction between both. In that subgroup, we did not find any significant difference between the radiological scans, which underlines the value of CBCT in the diagnosis of a bone affection.

All imaging modalities were found to have artifacts. CBCT and CT had a particularly high rate of artifacts. However, considering only artifacts that interfered with the evaluation of the tumor region, CBCT and MRI had significantly fewer of these than CT. This is in line with the present literature, in which more artifacts are reported for CT than for MRI [33, 35].

In addition, we see great advantages in the early diagnosis of bone erosion or invasion when a CBCT scan is performed during initial contact with a patient exhibiting OSCC or another oral malignancy. This allows the adjustment of the further staging examination and is usually not possible for CT and MRI. For example, it is possible to perform an angiography of the legs if a microvascular fibula transplant is considered or perform a preoperative percutaneous endoscopic gastrostomy. These can prevent delays in tumor therapy caused by a late diagnosis of a bone affection. It must be mentioned that CBCT is only an additional examination to CT and MRI, which are both necessary to visualize the tumor extent and to detect nodal and distant metastases [17]. Furthermore, current guidelines recommend dental diagnosis during staging examinations to prevent problems in cases of primary or adjuvant radiation therapy. This dental diagnosis is fully possible with a CBCT scan, and no further dental imaging is necessary [36].

Several limitations of our study must be considered. First, the evaluation of CBCT, CT, and MRI scans was performed by different persons (specialists in oral and maxillofacial surgery versus specialists in radiology). This could have caused observer bias that we tried to eliminate by having two specialists in consensus evaluate the images. However, we think this protocol reflects day-to-day practice quite accurately, as CBCT scans were normally evaluated by oral and maxillofacial surgeons, and CT and MRI scans by specialists in radiology. Another limitation is the fact that the CT scan protocols differed, and patients received, for example, CTs with different slice thicknesses. The development of new CBCT devices has led to an even higher resolution, such that the diagnostic accuracy of CBCT could be higher than depicted by the results of our study. Furthermore, the validity of the subgroup analysis was limited due to the small sample size. Only 45 patients were enrolled to evaluate small slice thicknesses in CT. This caused a high variability in the sensitivity, specificity, and accuracy values, and weakens the impact of our results. On the other hand, the study cohort in our subgroups is still larger than those in most of the others studies found in the literature. Furthermore, in 76 of 153 cases, no bone resection was performed. In these cases, the CBCT, CT, and MRI findings were validated by the follow-up. Such a follow-up is less accurate and cannot, for example, differentiate between a case of OSCC that primarily infiltrates the jawbone and has not been detected and OSCC that has grown secondarily during the six months of the follow-up into the jawbone. This limitation could have distorted our values. However, the sensitivities, specificities, and accuracies in the group with a histological examination of bone tissue (*n* = 77) were equal to those in the whole study collective.

Our data reveal a high diagnostic accuracy for all imaging modalities (CBCT, CT, and MRI) and support the routine use of CBCT scans in the staging examination of patients with oral malignancies and especially those with OSCC. Which combination of imaging modalities should be used must be investigated by further prospective studies.

## Conclusion

Our results support the use of CBCT in the detection of bone erosion or invasion in patients suffering from OSCC. We see great advantages in the early detection of a bone affection for the planning of further staging examinations and surgery. Therefore, CBCT should be considered at the point in time of initial contact with such patients.

## Data Availability

The dataset used to reach the conclusions in this article is included within the article. Further clinical data and information are not publicly available because other, currently unpublished, studies are based on it. However, these are available from the corresponding author upon reasonable request.
